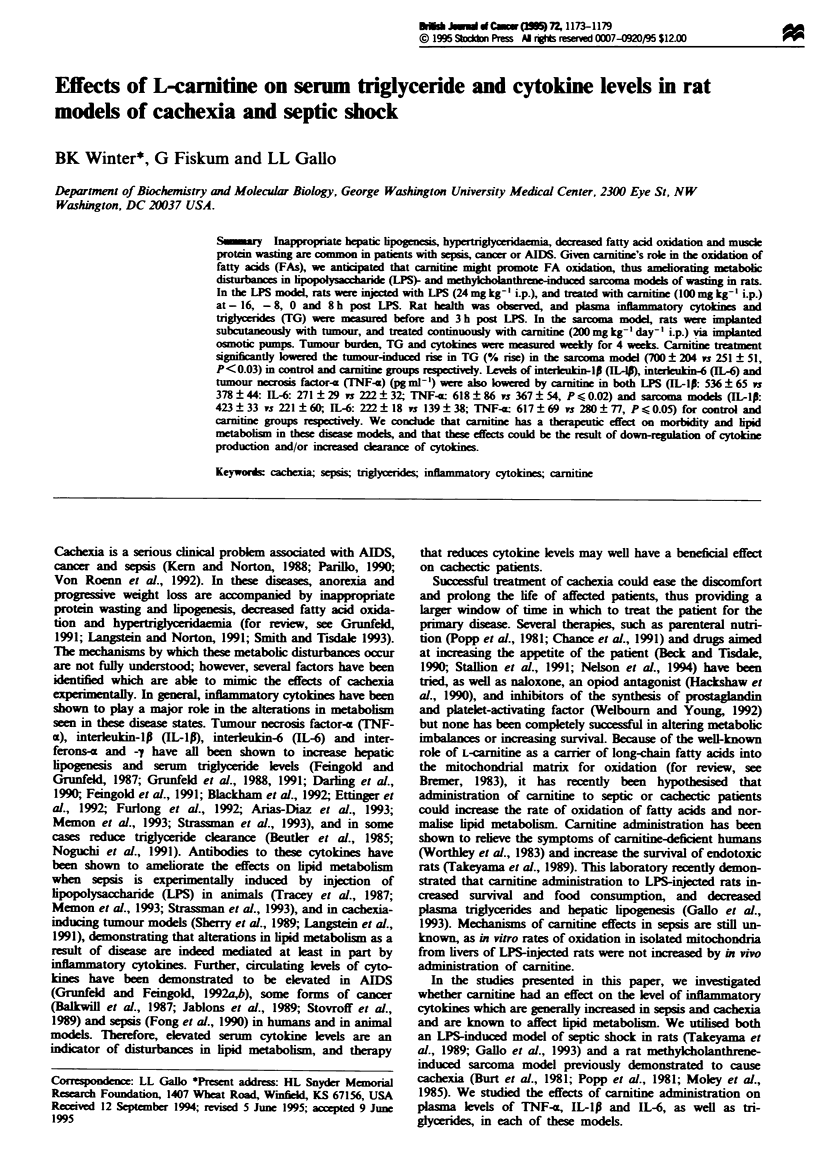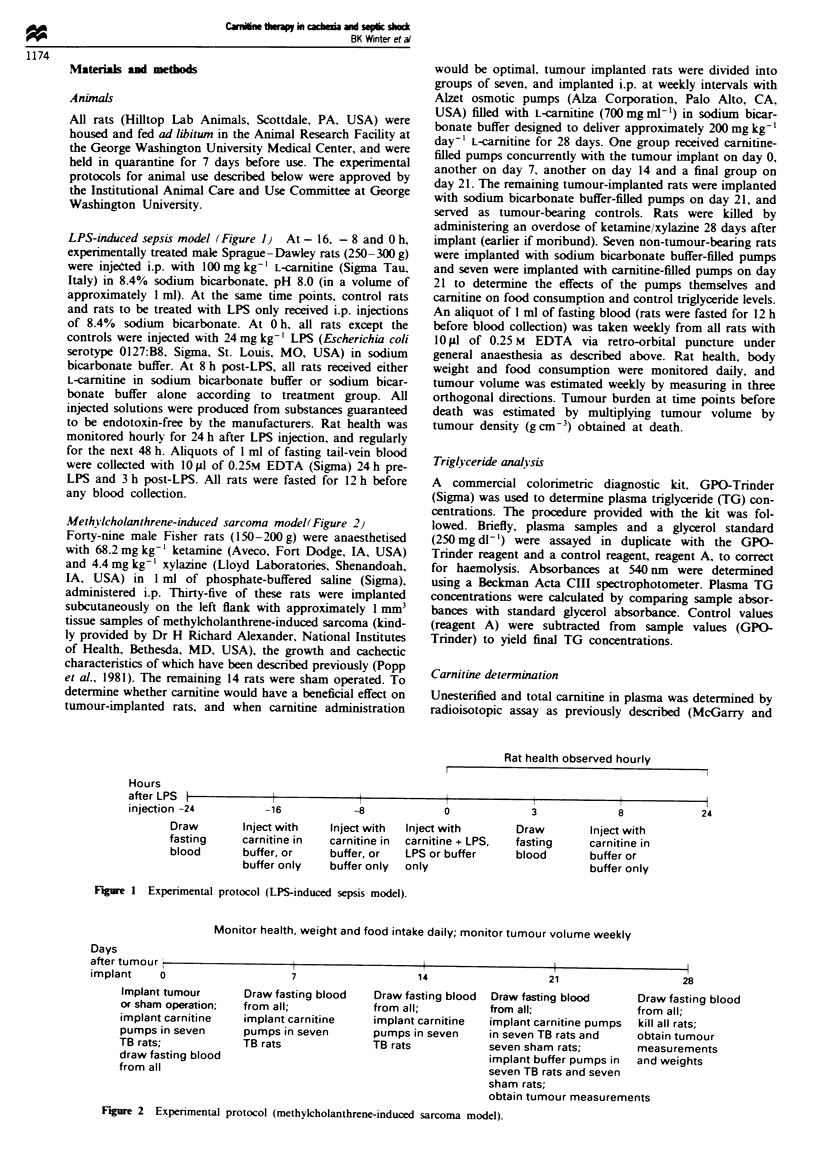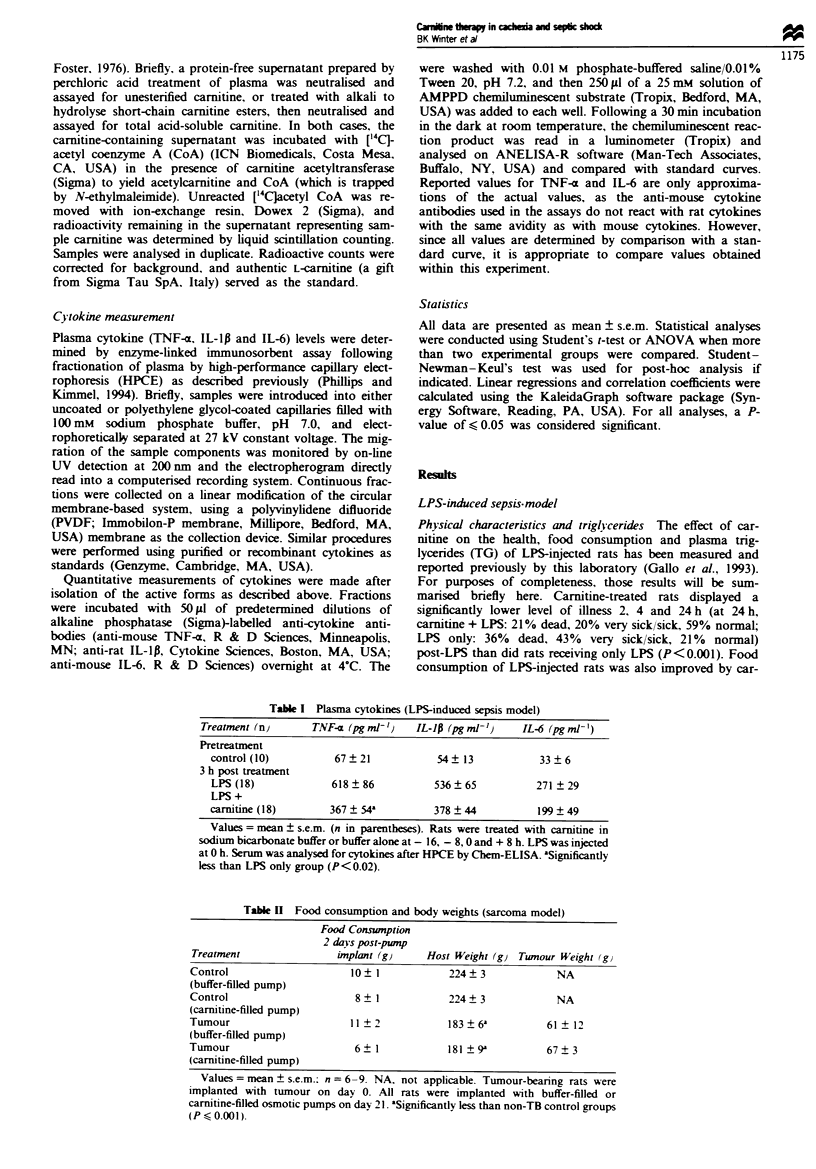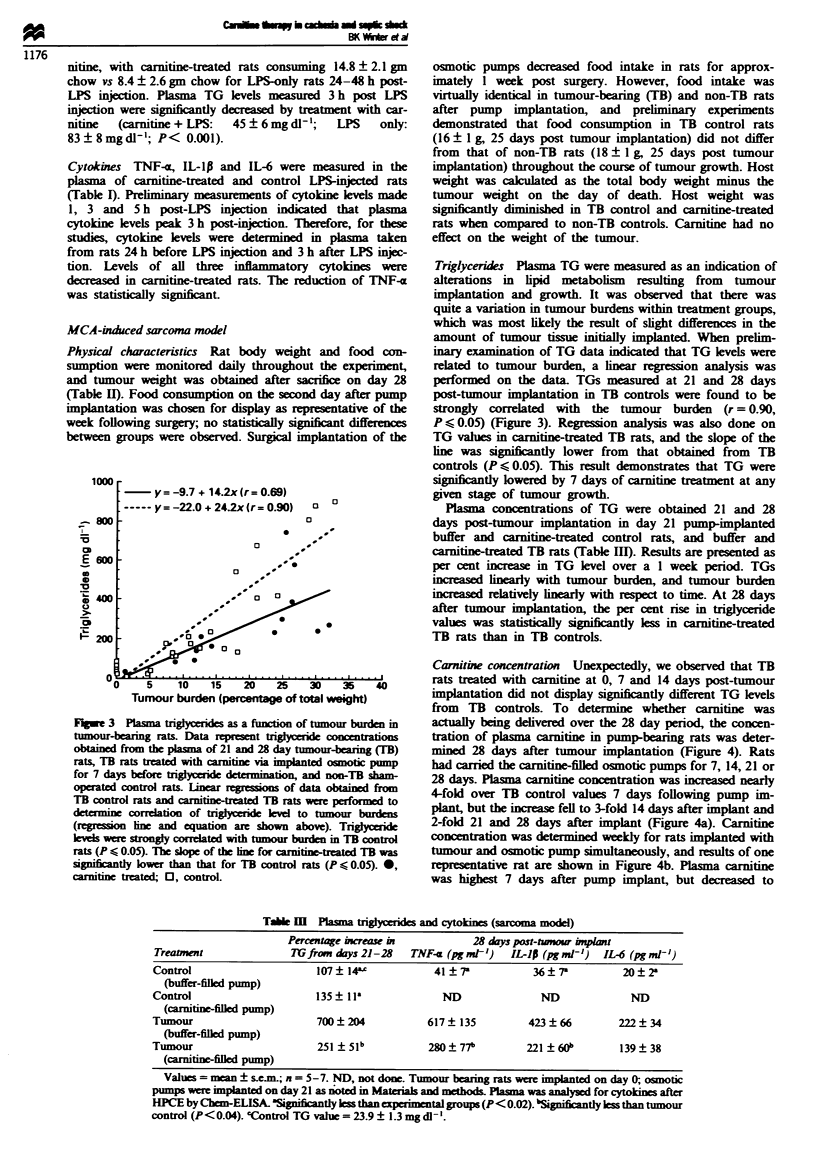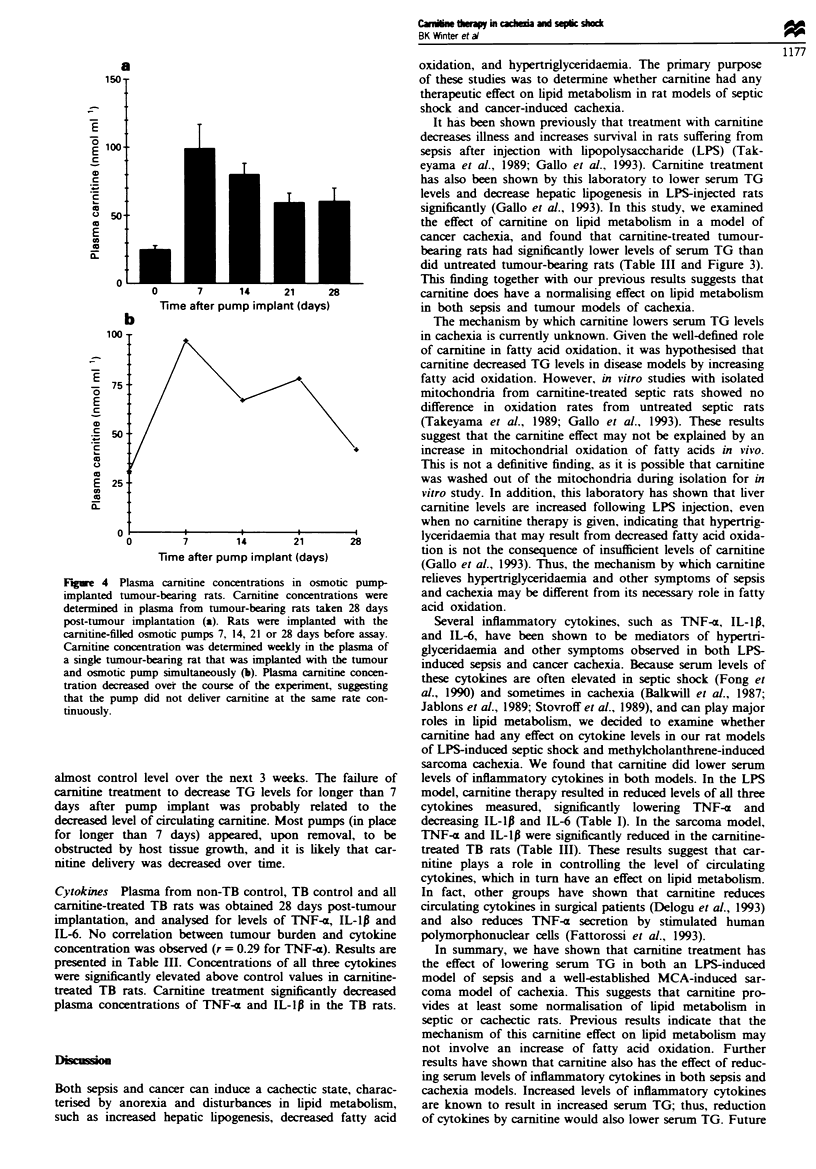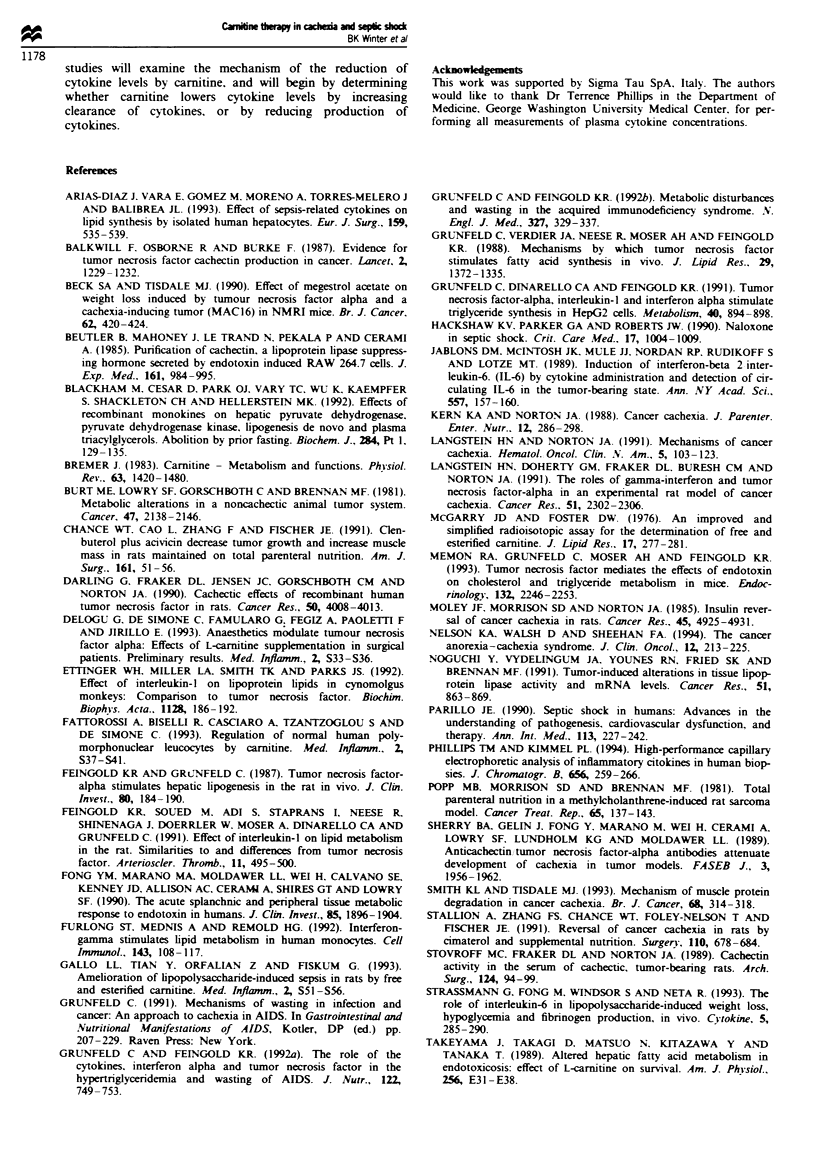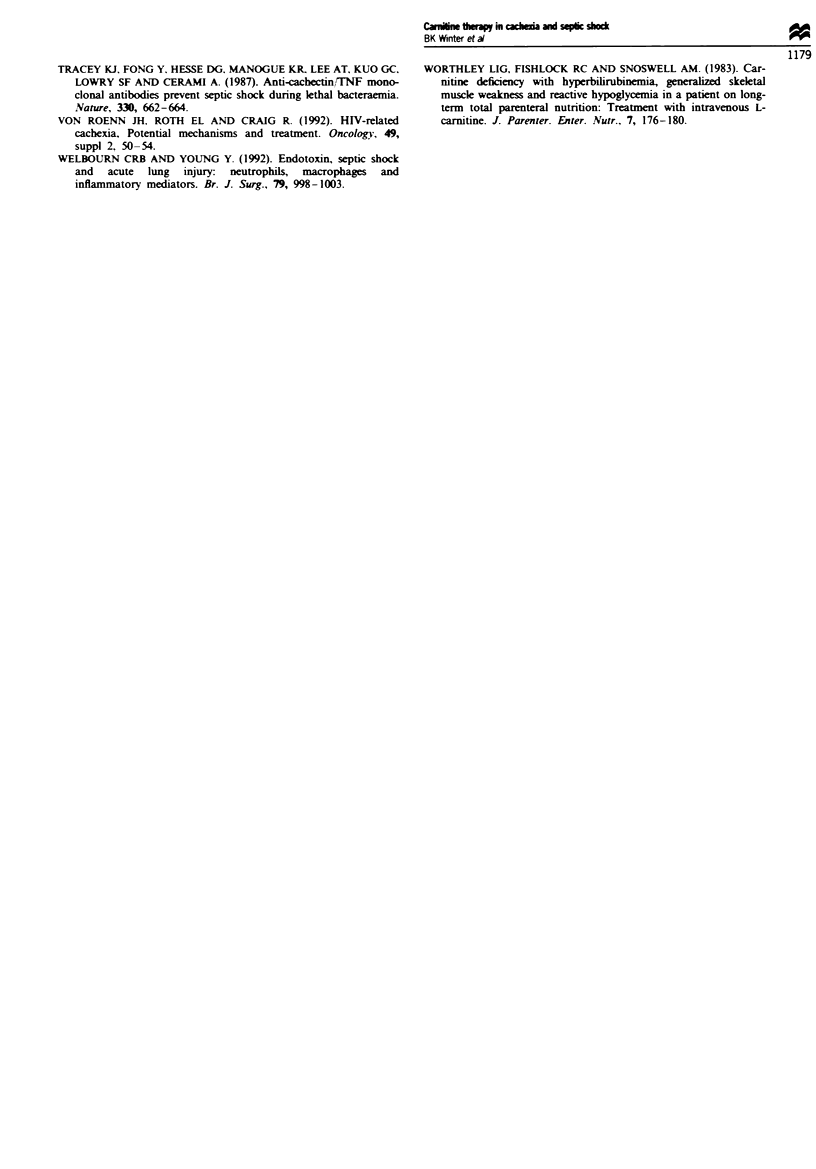# Effects of L-carnitine on serum triglyceride and cytokine levels in rat models of cachexia and septic shock.

**DOI:** 10.1038/bjc.1995.482

**Published:** 1995-11

**Authors:** B. K. Winter, G. Fiskum, L. L. Gallo

**Affiliations:** Department of Biochemistry and Molecular Biology, George Washington University Medical Center, Washington, DC 20037, USA.

## Abstract

Inappropriate hepatic lipogenesis, hypertriglyceridaemia, decreased fatty acid oxidation and muscle protein wasting are common in patients with sepsis, cancer or AIDS. Given carnitine's role in the oxidation of fatty acids (FAs), we anticipated that carnitine might promote FA oxidation, thus ameliorating metabolic disturbances in lipopolysaccharide (LPS)- and methylcholanthrene-induced sarcoma models of wasting in rats. In the LPS model, rats were injected with LPS (24 mg kg-1 i.p.), and treated with carnitine (100 mg kg-1 i.p.) at -16, -8, 0 and 8 h post LPS. Rat health was observed, and plasma inflammatory cytokines and triglycerides (TG) were measured before and 3 h post LPS. In the sarcoma model, rats were implanted subcutaneously with tumour, and treated continuously with carnitine (200 mg kg-1 day-1 i.p.) via implanted osmotic pumps. Tumour burden, TG and cytokines were measured weekly for 4 weeks. Carnitine treatment significantly lowered the tumour-induced rise in TG (% rise) in the sarcoma model (700 +/- 204 vs 251 +/- 51, P < 0.03) in control and carnitine groups respectively. Levels of interleukin-1 beta (IL-1 beta), interleukin-6 (IL-6) and tumour necrosis factor-alpha (TNF-alpha) (pg ml-1) were also lowered by carnitine in both LPS (IL-1 beta: 536 +/- 65 vs 378 +/- 44: IL-6: 271 +/- 29 vs 222 +/- 32; TNF-alpha: 618 +/- 86 vs 367 +/- 54, P < or = 0.02) and sarcoma models (IL-1 beta: 423 +/- 33 vs 221 +/- 60; IL-6: 222 +/- 18 vs 139 +/- 38; TNF-alpha: 617 +/- 69 vs 280 +/- 77, P < or = 0.05) for control and carnitine groups respectively. We conclude that carnitine has a therapeutic effect on morbidity and lipid metabolism in these disease models, and that these effects could be the result of down-regulation of cytokine production and/or increased clearance of cytokines.


© 1995 Stockton Press All rights reserved 0007–0920/95 $12.00

### Effects of L-carnitine on serum triglyceride and cytokine levels in rat models of cachexia and septic shock

BK Winter*, G Fiskum and LL Gallo

*Department of Biochemistry and Molecular Biology, George Washington University Medical Center, 2300 Eye St, NW Washington, DC 20037 USA.*

**Summary** Inappropriate hepatic lipogenesis, hypertriglyceridaemia, decreased fatty acid oxidation and muscle protein wasting are common in patients with sepsis, cancer or AIDS. Given carnitine's role in the oxidation of fatty acids (FAs), we anticipated that carnitine might promote FA oxidation, thus ameliorating metabolic disturbances in lipopolysaccharide (LPS)- and methylcholanthrene-induced sarcoma models of wasting in rats. In the LPS model, rats were injected with LPS (24 mg $kg^{-1}$ i.p.), and treated with carnitine (100 mg $kg^{-1}$ i.p.) at − 16, − 8, 0 and 8 h post LPS. Rat health was observed, and plasma inflammatory cytokines and triglycerides (TG) were measured before and 3 h post LPS. In the sarcoma model, rats were implanted subcutaneously with tumour, and treated continuously with carnitine (200 mg $kg^{-1}$ $day^{-1}$ i.p.) via implanted osmotic pumps. Tumour burden, TG and cytokines were measured weekly for 4 weeks. Carnitine treatment significantly lowered the tumour-induced rise in TG (% rise) in the sarcoma model (700 ± 204 *vs* 251 ± 51, $P<0.03$) in control and carnitine groups respectively. Levels of interleukin-1$\beta$ (IL-1$\beta$), interleukin-6 (IL-6) and tumour necrosis factor-$\alpha$ (TNF-$\alpha$) (pg $ml^{-1}$) were also lowered by carnitine in both LPS (IL-1$\beta$: 536 ± 65 *vs* 378 ± 44: IL-6: 271 ± 29 *vs* 222 ± 32; TNF-$\alpha$: 618 ± 86 *vs* 367 ± 54, $P \leqslant 0.02$) and sarcoma models (IL-1$\beta$: 423 ± 33 *vs* 221 ± 60; IL-6: 222 ± 18 *vs* 139 ± 38; TNF-$\alpha$: 617 ± 69 *vs* 280 ± 77, $P \leqslant 0.05$) for control and carnitine groups respectively. We conclude that carnitine has a therapeutic effect on morbidity and lipid metabolism in these disease models, and that these effects could be the result of down-regulation of cytokine production and/or increased clearance of cytokines.

**Keywords:** cachexia; sepsis; triglycerides; inflammatory cytokines; carnitine

Cachexia is a serious clinical problem associated with AIDS, cancer and sepsis (Kern and Norton, 1988; Parillo, 1990; Von Roenn *et al.*, 1992). In these diseases, anorexia and progressive weight loss are accompanied by inappropriate protein wasting and lipogenesis, decreased fatty acid oxidation and hypertriglyceridaemia (for review, see Grunfeld, 1991; Langstein and Norton, 1991; Smith and Tisdale 1993). The mechanisms by which these metabolic disturbances occur are not fully understood; however, several factors have been identified which are able to mimic the effects of cachexia experimentally. In general, inflammatory cytokines have been shown to play a major role in the alterations in metabolism seen in these disease states. Tumour necrosis factor-$\alpha$ (TNF-$\alpha$), interleukin-1$\beta$ (IL-1$\beta$), interleukin-6 (IL-6) and interferons-$\alpha$ and -$\gamma$ have all been shown to increase hepatic lipogenesis and serum triglyceride levels (Feingold and Grunfeld, 1987; Grunfeld *et al.*, 1988, 1991; Darling *et al.*, 1990; Feingold *et al.*, 1991; Blackham *et al.*, 1992; Ettinger *et al.*, 1992; Furlong *et al.*, 1992; Arias-Diaz *et al.*, 1993; Memon *et al.*, 1993; Strassman *et al.*, 1993), and in some cases reduce triglyceride clearance (Beutler *et al.*, 1985; Noguchi *et al.*, 1991). Antibodies to these cytokines have been shown to ameliorate the effects on lipid metabolism when sepsis is experimentally induced by injection of lipopolysaccharide (LPS) in animals (Tracey *et al.*, 1987; Memon *et al.*, 1993; Strassman *et al.*, 1993), and in cachexia-inducing tumour models (Sherry *et al.*, 1989; Langstein *et al.*, 1991), demonstrating that alterations in lipid metabolism as a result of disease are indeed mediated at least in part by inflammatory cytokines. Further, circulating levels of cytokines have been demonstrated to be elevated in AIDS (Grunfeld and Feingold, 1992*a,b*), some forms of cancer (Balkwill *et al.*, 1987; Jablons *et al.*, 1989; Stovroff *et al.*, 1989) and sepsis (Fong *et al.*, 1990) in humans and in animal models. Therefore, elevated serum cytokine levels are an indicator of disturbances in lipid metabolism, and therapy that reduces cytokine levels may well have a beneficial effect on cachectic patients.

Successful treatment of cachexia could ease the discomfort and prolong the life of affected patients, thus providing a larger window of time in which to treat the patient for the primary disease. Several therapies, such as parenteral nutrition (Popp *et al.*, 1981; Chance *et al.*, 1991) and drugs aimed at increasing the appetite of the patient (Beck and Tisdale, 1990; Stallion *et al.*, 1991; Nelson *et al.*, 1994) have been tried, as well as naloxone, an opiod antagonist (Hackshaw *et al.*, 1990), and inhibitors of the synthesis of prostaglandin and platelet-activating factor (Welbourn and Young, 1992) but none has been completely successful in altering metabolic imbalances or increasing survival. Because of the well-known role of L-carnitine as a carrier of long-chain fatty acids into the mitochondrial matrix for oxidation (for review, see Bremer, 1983), it has recently been hypothesised that administration of carnitine to septic or cachectic patients could increase the rate of oxidation of fatty acids and normalise lipid metabolism. Carnitine administration has been shown to relieve the symptoms of carnitine-deficient humans (Worthley *et al.*, 1983) and increase the survival of endotoxic rats (Takeyama *et al.*, 1989). This laboratory recently demonstrated that carnitine administration to LPS-injected rats increased survival and food consumption, and decreased plasma triglycerides and hepatic lipogenesis (Gallo *et al.*, 1993). Mechanisms of carnitine effects in sepsis are still unknown, as *in vitro* rates of oxidation in isolated mitochondria from livers of LPS-injected rats were not increased by *in vivo* administration of carnitine.

In the studies presented in this paper, we investigated whether carnitine had an effect on the level of inflammatory cytokines which are generally increased in sepsis and cachexia and are known to affect lipid metabolism. We utilised both an LPS-induced model of septic shock in rats (Takeyama *et al.*, 1989; Gallo *et al.*, 1993) and a rat methylcholanthrene-induced sarcoma model previously demonstrated to cause cachexia (Burt *et al.*, 1981; Popp *et al.*, 1981; Moley *et al.*, 1985). We studied the effects of carnitine administration on plasma levels of TNF-$\alpha$, IL-1$\beta$ and IL-6, as well as triglycerides, in each of these models.

Correspondence: LL Gallo *Present address: HL Snyder Memorial Research Foundation, 1407 Wheat Road, Winfield, KS 67156, USA

Received 12 September 1994; revised 5 June 1995; accepted 9 June 1995

### Materials and methods

### *Animals*

All rats (Hilltop Lab Animals, Scottdale, PA, USA) were housed and fed *ad libitum* in the Animal Research Facility at the George Washington University Medical Center, and were held in quarantine for 7 days before use. The experimental protocols for animal use described below were approved by the Institutional Animal Care and Use Committee at George Washington University.

*LPS-induced sepsis model (Figure 1)* At − 16, − 8 and 0 h, experimentally treated male Sprague–Dawley rats (250–300 g) were injected i.p. with 100 mg $kg^{-1}$ L-carnitine (Sigma Tau, Italy) in 8.4% sodium bicarbonate, pH 8.0 (in a volume of approximately 1 ml). At the same time points, control rats and rats to be treated with LPS only received i.p. injections of 8.4% sodium bicarbonate. At 0 h, all rats except the controls were injected with 24 mg $kg^{-1}$ LPS (*Escherichia coli* serotype 0127:B8, Sigma, St. Louis, MO, USA) in sodium bicarbonate buffer. At 8 h post-LPS, all rats received either L-carnitine in sodium bicarbonate buffer or sodium bicarbonate buffer alone according to treatment group. All injected solutions were produced from substances guaranteed to be endotoxin-free by the manufacturers. Rat health was monitored hourly for 24 h after LPS injection, and regularly for the next 48 h. Aliquots of 1 ml of fasting tail-vein blood were collected with 10 µl of 0.25M EDTA (Sigma) 24 h pre-LPS and 3 h post-LPS. All rats were fasted for 12 h before any blood collection.

*Methylcholanthrene-induced sarcoma model (Figure 2)* Forty-nine male Fisher rats (150–200 g) were anaesthetised with 68.2 mg $kg^{-1}$ ketamine (Aveco, Fort Dodge, IA, USA) and 4.4 mg $kg^{-1}$ xylazine (Lloyd Laboratories, Shenandoah, IA, USA) in 1 ml of phosphate-buffered saline (Sigma), administered i.p. Thirty-five of these rats were implanted subcutaneously on the left flank with approximately 1 $mm^3$ tissue samples of methylcholanthrene-induced sarcoma (kindly provided by Dr H Richard Alexander, National Institutes of Health, Bethesda, MD, USA), the growth and cachectic characteristics of which have been described previously (Popp *et al.*, 1981). The remaining 14 rats were sham operated. To determine whether carnitine would have a beneficial effect on tumour-implanted rats, and when carnitine administration would be optimal, tumour implanted rats were divided into groups of seven, and implanted i.p. at weekly intervals with Alzet osmotic pumps (Alza Corporation, Palo Alto, CA, USA) filled with L-carnitine (700 mg $ml^{-1}$) in sodium bicarbonate buffer designed to deliver approximately 200 mg $kg^{-1}$ $day^{-1}$ L-carnitine for 28 days. One group received carnitine-filled pumps concurrently with the tumour implant on day 0, another on day 7, another on day 14 and a final group on day 21. The remaining tumour-implanted rats were implanted with sodium bicarbonate buffer-filled pumps on day 21, and served as tumour-bearing controls. Rats were killed by administering an overdose of ketamine/xylazine 28 days after implant (earlier if moribund). Seven non-tumour-bearing rats were implanted with sodium bicarbonate buffer-filled pumps and seven were implanted with carnitine-filled pumps on day 21 to determine the effects of the pumps themselves and carnitine on food consumption and control triglyceride levels. An aliquot of 1 ml of fasting blood (rats were fasted for 12 h before blood collection) was taken weekly from all rats with 10 µl of 0.25 M EDTA via retro-orbital puncture under general anaesthesia as described above. Rat health, body weight and food consumption were monitored daily, and tumour volume was estimated weekly by measuring in three orthogonal directions. Tumour burden at time points before death was estimated by multiplying tumour volume by tumour density (g $cm^{-3}$) obtained at death.

### *Triglyceride analysis*

A commercial colorimetric diagnostic kit, GPO-Trinder (Sigma) was used to determine plasma triglyceride (TG) concentrations. The procedure provided with the kit was followed. Briefly, plasma samples and a glycerol standard (250 mg $dl^{-1}$) were assayed in duplicate with the GPO-Trinder reagent and a control reagent, reagent A, to correct for haemolysis. Absorbances at 540 nm were determined using a Beckman Acta CIII spectrophotometer. Plasma TG concentrations were calculated by comparing sample absorbances with standard glycerol absorbance. Control values (reagent A) were subtracted from sample values (GPO-Trinder) to yield final TG concentrations.

### *Carnitine determination*

Unesterified and total carnitine in plasma was determined by radioisotopic assay as previously described (McGarry and

**Figure 1** Experimental protocol (LPS-induced sepsis model).

**Figure 2** Experimental protocol (methylcholanthrene-induced sarcoma model).

Foster, 1976). Briefly, a protein-free supernatant prepared by perchloric acid treatment of plasma was neutralised and assayed for unesterified carnitine, or treated with alkali to hydrolyse short-chain carnitine esters, then neutralised and assayed for total acid-soluble carnitine. In both cases, the carnitine-containing supernatant was incubated with [$^{14}$C]-acetyl coenzyme A (CoA) (ICN Biomedicals, Costa Mesa, CA, USA) in the presence of carnitine acetyltransferase (Sigma) to yield acetylcarnitine and CoA (which is trapped by *N*-ethylmaleimide). Unreacted [$^{14}$C]acetyl CoA was removed with ion-exchange resin, Dowex 2 (Sigma), and radioactivity remaining in the supernatant representing sample carnitine was determined by liquid scintillation counting. Samples were analysed in duplicate. Radioactive counts were corrected for background, and authentic L-carnitine (a gift from Sigma Tau SpA, Italy) served as the standard.

### *Cytokine measurement*

Plasma cytokine (TNF-α, IL-1β and IL-6) levels were determined by enzyme-linked immunosorbent assay following fractionation of plasma by high-performance capillary electrophoresis (HPCE) as described previously (Phillips and Kimmel, 1994). Briefly, samples were introduced into either uncoated or polyethylene glycol-coated capillaries filled with 100 mM sodium phosphate buffer, pH 7.0, and electrophoretically separated at 27 kV constant voltage. The migration of the sample components was monitored by on-line UV detection at 200 nm and the electropherogram directly read into a computerised recording system. Continuous fractions were collected on a linear modification of the circular membrane-based system, using a polyvinylidene difluoride (PVDF; Immobilon-P membrane, Millipore, Bedford, MA, USA) membrane as the collection device. Similar procedures were performed using purified or recombinant cytokines as standards (Genzyme, Cambridge, MA, USA).

Quantitative measurements of cytokines were made after isolation of the active forms as described above. Fractions were incubated with 50 μl of predetermined dilutions of alkaline phosphatase (Sigma)-labelled anti-cytokine antibodies (anti-mouse TNF-α, R & D Sciences, Minneapolis, MN; anti-rat IL-1β, Cytokine Sciences, Boston, MA, USA; anti-mouse IL-6, R & D Sciences) overnight at 4°C. The were washed with 0.01 M phosphate-buffered saline/0.01% Tween 20, pH 7.2, and then 250 μl of a 25 mM solution of AMPPD chemiluminescent substrate (Tropix, Bedford, MA, USA) was added to each well. Following a 30 min incubation in the dark at room temperature, the chemiluminescent reaction product was read in a luminometer (Tropix) and analysed on ANELISA-R software (Man-Tech Associates, Buffalo, NY, USA) and compared with standard curves. Reported values for TNF-α and IL-6 are only approximations of the actual values, as the anti-mouse cytokine antibodies used in the assays do not react with rat cytokines with the same avidity as with mouse cytokines. However, since all values are determined by comparison with a standard curve, it is appropriate to compare values obtained within this experiment.

### *Statistics*

All data are presented as mean ± s.e.m. Statistical analyses were conducted using Student's *t*-test or ANOVA when more than two experimental groups were compared. Student–Newman–Keul's test was used for post-hoc analysis if indicated. Linear regressions and correlation coefficients were calculated using the KaleidaGraph software package (Synergy Software, Reading, PA, USA). For all analyses, a *P*-value of ≤ 0.05 was considered significant.

### Results

### *LPS-induced sepsis model*

*Physical characteristics and triglycerides* The effect of carnitine on the health, food consumption and plasma triglycerides (TG) of LPS-injected rats has been measured and reported previously by this laboratory (Gallo *et al.*, 1993). For purposes of completeness, those results will be summarised briefly here. Carnitine-treated rats displayed a significantly lower level of illness 2, 4 and 24 h (at 24 h, carnitine + LPS: 21% dead, 20% very sick/sick, 59% normal; LPS only: 36% dead, 43% very sick/sick, 21% normal) post-LPS than did rats receiving only LPS ($P < 0.001$). Food consumption of LPS-injected rats was also improved by car-

**Table I** Plasma cytokines (LPS-induced sepsis model)

| *Treatment* (n) | *TNF-α (pg ml$^{-1}$)* | *IL-1β (pg ml$^{-1}$)* | *IL-6 (pg ml$^{-1}$)* |
|---|---|---|---|
| Pretreatment control (10) | 67 ± 21 | 54 ± 13 | 33 ± 6 |
| 3 h post treatment | | | |
| LPS (18) | 618 ± 86 | 536 ± 65 | 271 ± 29 |
| LPS + carnitine (18) | 367 ± 54[a] | 378 ± 44 | 199 ± 49 |

Values = mean ± s.e.m. (*n* in parentheses). Rats were treated with carnitine in sodium bicarbonate buffer or buffer alone at − 16, − 8, 0 and + 8 h. LPS was injected at 0 h. Serum was analysed for cytokines after HPCE by Chem-ELISA. [a]Significantly less than LPS only group ($P < 0.02$).

**Table II** Food consumption and body weights (sarcoma model)

| *Treatment* | *Food Consumption 2 days post-pump implant (g)* | *Host Weight (g)* | *Tumour Weight (g)* |
|---|---|---|---|
| Control (buffer-filled pump) | 10 ± 1 | 224 ± 3 | NA |
| Control (carnitine-filled pump) | 8 ± 1 | 224 ± 3 | NA |
| Tumour (buffer-filled pump) | 11 ± 2 | 183 ± 6[a] | 61 ± 12 |
| Tumour (carnitine-filled pump) | 6 ± 1 | 181 ± 9[a] | 67 ± 3 |

Values = mean ± s.e.m.; *n* = 6–9. NA, not applicable. Tumour-bearing rats were implanted with tumour on day 0. All rats were implanted with buffer-filled or carnitine-filled osmotic pumps on day 21. [a]Significantly less than non-TB control groups ($P \leq 0.001$).

nitine, with carnitine-treated rats consuming 14.8 ± 2.1 gm chow *vs* 8.4 ± 2.6 gm chow for LPS-only rats 24–48 h post-LPS injection. Plasma TG levels measured 3 h post LPS injection were significantly decreased by treatment with carnitine (carnitine + LPS: 45 ± 6 mg $dl^{-1}$; LPS only: 83 ± 8 mg $dl^{-1}$; $P < 0.001$).

*Cytokines* TNF-α, IL-1β and IL-6 were measured in the plasma of carnitine-treated and control LPS-injected rats (Table I). Preliminary measurements of cytokine levels made 1, 3 and 5 h post-LPS injection indicated that plasma cytokine levels peak 3 h post-injection. Therefore, for these studies, cytokine levels were determined in plasma taken from rats 24 h before LPS injection and 3 h after LPS injection. Levels of all three inflammatory cytokines were decreased in carnitine-treated rats. The reduction of TNF-α was statistically significant.

### *MCA-induced sarcoma model*

*Physical characteristics* Rat body weight and food consumption were monitored daily throughout the experiment, and tumour weight was obtained after sacrifice on day 28 (Table II). Food consumption on the second day after pump implantation was chosen for display as representative of the week following surgery; no statistically significant differences between groups were observed. Surgical implantation of the osmotic pumps decreased food intake in rats for approximately 1 week post surgery. However, food intake was virtually identical in tumour-bearing (TB) and non-TB rats after pump implantation, and preliminary experiments demonstrated that food consumption in TB control rats (16 ± 1 g, 25 days post tumour implantation) did not differ from that of non-TB rats (18 ± 1 g, 25 days post tumour implantation) throughout the course of tumour growth. Host weight was calculated as the total body weight minus the tumour weight on the day of death. Host weight was significantly diminished in TB control and carnitine-treated rats when compared to non-TB controls. Carnitine had no effect on the weight of the tumour.

**Figure 3** Plasma triglycerides as a function of tumour burden in tumour-bearing rats. Data represent triglyceride concentrations obtained from the plasma of 21 and 28 day tumour-bearing (TB) rats, TB rats treated with carnitine via implanted osmotic pump for 7 days before triglyceride determination, and non-TB sham-operated control rats. Linear regressions of data obtained from TB control rats and carnitine-treated TB rats were performed to determine correlation of triglyceride level to tumour burdens (regression line and equation are shown above). Triglyceride levels were strongly correlated with tumour burden in TB control rats ($P \leq 0.05$). The slope of the line for carnitine-treated TB was significantly lower than that for TB control rats ($P \leq 0.05$). ●, carnitine treated; □, control.

*Triglycerides* Plasma TG were measured as an indication of alterations in lipid metabolism resulting from tumour implantation and growth. It was observed that there was quite a variation in tumour burdens within treatment groups, which was most likely the result of slight differences in the amount of tumour tissue initially implanted. When preliminary examination of TG data indicated that TG levels were related to tumour burden, a linear regression analysis was performed on the data. TGs measured at 21 and 28 days post-tumour implantation in TB controls were found to be strongly correlated with the tumour burden ($r = 0.90$, $P \leq 0.05$) (Figure 3). Regression analysis was also done on TG values in carnitine-treated TB rats, and the slope of the line was significantly lower from that obtained from TB controls ($P \leq 0.05$). This result demonstrates that TG were significantly lowered by 7 days of carnitine treatment at any given stage of tumour growth.

Plasma concentrations of TG were obtained 21 and 28 days post-tumour implantation in day 21 pump-implanted buffer and carnitine-treated control rats, and buffer and carnitine-treated TB rats (Table III). Results are presented as per cent increase in TG level over a 1 week period. TGs increased linearly with tumour burden, and tumour burden increased relatively linearly with respect to time. At 28 days after tumour implantation, the per cent rise in triglyceride values was statistically significantly less in carnitine-treated TB rats than in TB controls.

*Carnitine concentration* Unexpectedly, we observed that TB rats treated with carnitine at 0, 7 and 14 days post-tumour implantation did not display significantly different TG levels from TB controls. To determine whether carnitine was actually being delivered over the 28 day period, the concentration of plasma carnitine in pump-bearing rats was determined 28 days after tumour implantation (Figure 4). Rats had carried the carnitine-filled osmotic pumps for 7, 14, 21 or 28 days. Plasma carnitine concentration was increased nearly 4-fold over TB control values 7 days following pump implant, but the increase fell to 3-fold 14 days after implant and 2-fold 21 and 28 days after implant (Figure 4a). Carnitine concentration was determined weekly for rats implanted with tumour and osmotic pump simultaneously, and results of one representative rat are shown in Figure 4b. Plasma carnitine was highest 7 days after pump implant, but decreased to

**Table III** Plasma triglycerides and cytokines (sarcoma model)

| *Treatment* | *Percentage increase in TG from days 21–28* | *28 days post-tumour implant* TNF-α ($pg\ ml^{-1}$) | IL-1β ($pg\ ml^{-1}$) | IL-6 ($pg\ ml^{-1}$) |
|---|---|---|---|---|
| Control (buffer-filled pump) | 107 ± 14[a,c] | 41 ± 7[a] | 36 ± 7[a] | 20 ± 2[a] |
| Control (carnitine-filled pump) | 135 ± 11[a] | ND | ND | ND |
| Tumour (buffer-filled pump) | 700 ± 204 | 617 ± 135 | 423 ± 66 | 222 ± 34 |
| Tumour (carnitine-filled pump) | 251 ± 51[b] | 280 ± 77[b] | 221 ± 60[b] | 139 ± 38 |

Values = mean ± s.e.m.; $n = 5–7$. ND, not done. Tumour bearing rats were implanted on day 0; osmotic pumps were implanted on day 21 as noted in Materials and methods. Plasma was analysed for cytokines after HPCE by Chem-ELISA. [a]Significantly less than experimental groups ($P < 0.02$). [b]Significantly less than tumour control ($P < 0.04$). [c]Control TG value = 23.9 ± 1.3 mg $dl^{-1}$.

**Figure 4** Plasma carnitine concentrations in osmotic pump-implanted tumour-bearing rats. Carnitine concentrations were determined in plasma from tumour-bearing rats taken 28 days post-tumour implantation (**a**). Rats were implanted with the carnitine-filled osmotic pumps 7, 14, 21 or 28 days before assay. Carnitine concentration was determined weekly in the plasma of a single tumour-bearing rat that was implanted with the tumour and osmotic pump simultaneously (**b**). Plasma carnitine concentration decreased over the course of the experiment, suggesting that the pump did not deliver carnitine at the same rate continuously.

almost control level over the next 3 weeks. The failure of carnitine treatment to decrease TG levels for longer than 7 days after pump implant was probably related to the decreased level of circulating carnitine. Most pumps (in place for longer than 7 days) appeared, upon removal, to be obstructed by host tissue growth, and it is likely that carnitine delivery was decreased over time.

*Cytokines* Plasma from non-TB control, TB control and all carnitine-treated TB rats was obtained 28 days post-tumour implantation, and analysed for levels of TNF-α, IL-1β and IL-6. No correlation between tumour burden and cytokine concentration was observed ($r = 0.29$ for TNF-α). Results are presented in Table III. Concentrations of all three cytokines were significantly elevated above control values in carnitine-treated TB rats. Carnitine treatment significantly decreased plasma concentrations of TNF-α and IL-1β in the TB rats.

### Discussion

Both sepsis and cancer can induce a cachectic state, characterised by anorexia and disturbances in lipid metabolism, such as increased hepatic lipogenesis, decreased fatty acid oxidation, and hypertriglyceridaemia. The primary purpose of these studies was to determine whether carnitine had any therapeutic effect on lipid metabolism in rat models of septic shock and cancer-induced cachexia.

It has been shown previously that treatment with carnitine decreases illness and increases survival in rats suffering from sepsis after injection with lipopolysaccharide (LPS) (Takeyama *et al*., 1989; Gallo *et al*., 1993). Carnitine treatment has also been shown by this laboratory to lower serum TG levels and decrease hepatic lipogenesis in LPS-injected rats significantly (Gallo *et al*., 1993). In this study, we examined the effect of carnitine on lipid metabolism in a model of cancer cachexia, and found that carnitine-treated tumour-bearing rats had significantly lower levels of serum TG than did untreated tumour-bearing rats (Table III and Figure 3). This finding together with our previous results suggests that carnitine does have a normalising effect on lipid metabolism in both sepsis and tumour models of cachexia.

The mechanism by which carnitine lowers serum TG levels in cachexia is currently unknown. Given the well-defined role of carnitine in fatty acid oxidation, it was hypothesised that carnitine decreased TG levels in disease models by increasing fatty acid oxidation. However, *in vitro* studies with isolated mitochondria from carnitine-treated septic rats showed no difference in oxidation rates from untreated septic rats (Takeyama *et al*., 1989; Gallo *et al*., 1993). These results suggest that the carnitine effect may not be explained by an increase in mitochondrial oxidation of fatty acids *in vivo*. This is not a definitive finding, as it is possible that carnitine was washed out of the mitochondria during isolation for *in vitro* study. In addition, this laboratory has shown that liver carnitine levels are increased following LPS injection, even when no carnitine therapy is given, indicating that hypertriglyceridaemia that may result from decreased fatty acid oxidation is not the consequence of insufficient levels of carnitine (Gallo *et al*., 1993). Thus, the mechanism by which carnitine relieves hypertriglyceridaemia and other symptoms of sepsis and cachexia may be different from its necessary role in fatty acid oxidation.

Several inflammatory cytokines, such as TNF-α, IL-1β, and IL-6, have been shown to be mediators of hypertriglyceridaemia and other symptoms observed in both LPS-induced sepsis and cancer cachexia. Because serum levels of these cytokines are often elevated in septic shock (Fong *et al*., 1990) and sometimes in cachexia (Balkwill *et al*., 1987; Jablons *et al*., 1989; Stovroff *et al*., 1989), and can play major roles in lipid metabolism, we decided to examine whether carnitine had any effect on cytokine levels in our rat models of LPS-induced septic shock and methylcholanthrene-induced sarcoma cachexia. We found that carnitine did lower serum levels of inflammatory cytokines in both models. In the LPS model, carnitine therapy resulted in reduced levels of all three cytokines measured, significantly lowering TNF-α and decreasing IL-1β and IL-6 (Table I). In the sarcoma model, TNF-α and IL-1β were significantly reduced in the carnitine-treated TB rats (Table III). These results suggest that carnitine plays a role in controlling the level of circulating cytokines, which in turn have an effect on lipid metabolism. In fact, other groups have shown that carnitine reduces circulating cytokines in surgical patients (Delogu *et al*., 1993) and also reduces TNF-α secretion by stimulated human polymorphonuclear cells (Fattorossi *et al*., 1993).

In summary, we have shown that carnitine treatment has the effect of lowering serum TG in both an LPS-induced model of sepsis and a well-established MCA-induced sarcoma model of cachexia. This suggests that carnitine provides at least some normalisation of lipid metabolism in septic or cachectic rats. Previous results indicate that the mechanism of this carnitine effect on lipid metabolism may not involve an increase of fatty acid oxidation. Further results have shown that carnitine also has the effect of reducing serum levels of inflammatory cytokines in both sepsis and cachexia models. Increased levels of inflammatory cytokines are known to result in increased serum TG; thus, reduction of cytokines by carnitine would also lower serum TG. Future studies will examine the mechanism of the reduction of cytokine levels by carnitine, and will begin by determining whether carnitine lowers cytokine levels by increasing clearance of cytokines, or by reducing production of cytokines.

**Acknowledgements**
This work was supported by Sigma Tau SpA, Italy. The authors would like to thank Dr Terrence Phillips in the Department of Medicine, George Washington University Medical Center, for performing all measurements of plasma cytokine concentrations.

**References**

ARIAS-DIAZ J, VARA E, GOMEZ M, MORENO A, TORRES-MELERO J AND BALIBREA JL. (1993). Effect of sepsis-related cytokines on lipid synthesis by isolated human hepatocytes. *Eur. J. Surg.*, **159**, 535-539.

BALKWILL F, OSBORNE R AND BURKE F. (1987). Evidence for tumor necrosis factor cachectin production in cancer. *Lancet*, **2**, 1229-1232.

BECK SA AND TISDALE MJ. (1990). Effect of megestrol acetate on weight loss induced by tumour necrosis factor alpha and a cachexia-inducing tumor (MAC16) in NMRI mice. *Br. J. Cancer*, **62**, 420-424.

BEUTLER B, MAHONEY J, LE TRAND N, PEKALA P AND CERAMI A. (1985). Purification of cachectin, a lipoprotein lipase suppressing hormone secreted by endotoxin induced RAW 264.7 cells. *J. Exp. Med.*, **161**, 984-995.

BLACKHAM M, CESAR D, PARK OJ, VARY TC, WU K, KAEMPFER S, SHACKLETON CH AND HELLERSTEIN MK. (1992). Effects of recombinant monokines on hepatic pyruvate dehydrogenase, pyruvate dehydrogenase kinase, lipogenesis de novo and plasma triacylglycerols. Abolition by prior fasting. *Biochem. J.*, **284**, Pt 1, 129-135.

BREMER J. (1983). Carnitine – Metabolism and functions. *Physiol. Rev.*, **63**, 1420-1480.

BURT ME, LOWRY SF, GORSCHBOTH C AND BRENNAN MF. (1981). Metabolic alterations in a noncachectic animal tumor system. *Cancer*, **47**, 2138-2146.

CHANCE WT, CAO L, ZHANG F AND FISCHER JE. (1991). Clenbuterol plus acivicin decrease tumor growth and increase muscle mass in rats maintained on total parenteral nutrition. *Am. J. Surg.*, **161**, 51-56.

DARLING G, FRAKER DL, JENSEN JC, GORSCHBOTH CM AND NORTON JA. (1990). Cachectic effects of recombinant human tumor necrosis factor in rats. *Cancer Res.*, **50**, 4008-4013.

DELOGU G, DE SIMONE C, FAMULARO G, FEGIZ A, PAOLETTI F AND JIRILLO E. (1993). Anaesthetics modulate tumour necrosis factor alpha: Effects of L-carnitine supplementation in surgical patients. Preliminary results. *Med. Inflamm.*, **2**, S33-S36.

ETTINGER WH, MILLER LA, SMITH TK AND PARKS JS. (1992). Effect of interleukin-1 on lipoprotein lipids in cynomolgus monkeys: Comparison to tumor necrosis factor. *Biochim. Biophys. Acta.*, **1128**, 186-192.

FATTOROSSI A, BISELLI R, CASCIARO A, TZANTZOGLOU S AND DE SIMONE C. (1993). Regulation of normal human polymorphonuclear leucocytes by carnitine. *Med. Inflamm.*, **2**, S37-S41.

FEINGOLD KR AND GRUNFELD C. (1987). Tumor necrosis factor-alpha stimulates hepatic lipogenesis in the rat in vivo. *J. Clin. Invest.*, **80**, 184-190.

FEINGOLD KR, SOUED M, ADI S, STAPRANS I, NEESE R, SHINENAGA J, DOERRLER W, MOSER A, DINARELLO CA AND GRUNFELD C. (1991). Effect of interleukin-1 on lipid metabolism in the rat. Similarities to and differences from tumor necrosis factor. *Arterioscler. Thromb.*, **11**, 495-500.

FONG YM, MARANO MA, MOLDAWER LL, WEI H, CALVANO SE, KENNEY JD, ALLISON AC, CERAMI A, SHIRES GT AND LOWRY SF. (1990). The acute splanchnic and peripheral tissue metabolic response to endotoxin in humans. *J. Clin. Invest.*, **85**, 1896-1904.

FURLONG ST, MEDNIS A AND REMOLD HG. (1992). Interferon-gamma stimulates lipid metabolism in human monocytes. *Cell Immunol.*, **143**, 108-117.

GALLO LL, TIAN Y, ORFALIAN Z AND FISKUM G. (1993). Amelioration of lipopolysaccharide-induced sepsis in rats by free and esterified carnitine. *Med. Inflamm.*, **2**, S51-S56.

GRUNFELD C. (1991). Mechanisms of wasting in infection and cancer: An approach to cachexia in AIDS. In *Gastrointestinal and Nutritional Manifestations of AIDS*, Kotler, DP (ed.) pp. 207-229. Raven Press: New York.

GRUNFELD C AND FEINGOLD KR. (1992*a*). The role of the cytokines, interferon alpha and tumor necrosis factor in the hypertriglyceridemia and wasting of AIDS. *J. Nutr.*, **122**, 749-753.

GRUNFELD C AND FEINGOLD KR. (1992*b*). Metabolic disturbances and wasting in the acquired immunodeficiency syndrome. *N. Engl. J. Med.*, **327**, 329-337.

GRUNFELD C, VERDIER JA, NEESE R, MOSER AH AND FEINGOLD KR. (1988). Mechanisms by which tumor necrosis factor stimulates fatty acid synthesis in vivo. *J. Lipid Res.*, **29**, 1372-1335.

GRUNFELD C, DINARELLO CA AND FEINGOLD KR. (1991). Tumor necrosis factor-alpha, interleukin-1 and interferon alpha stimulate triglyceride synthesis in HepG2 cells. *Metabolism*, **40**, 894-898.

HACKSHAW KV, PARKER GA AND ROBERTS JW. (1990). Naloxone in septic shock. *Crit. Care Med.*, **17**, 1004-1009.

JABLONS DM, MCINTOSH JK, MULE JJ, NORDAN RP, RUDIKOFF S AND LOTZE MT. (1989). Induction of interferon-beta 2 interleukin-6. (IL-6) by cytokine administration and detection of circulating IL-6 in the tumor-bearing state. *Ann. NY Acad. Sci.*, **557**, 157-160.

KERN KA AND NORTON JA. (1988). Cancer cachexia. *J. Parenter. Enter. Nutr.*, **12**, 286-298.

LANGSTEIN HN AND NORTON JA. (1991). Mechanisms of cancer cachexia. *Hematol. Oncol. Clin. N. Am.*, **5**, 103-123.

LANGSTEIN HN, DOHERTY GM, FRAKER DL, BURESH CM AND NORTON JA. (1991). The roles of gamma-interferon and tumor necrosis factor-alpha in an experimental rat model of cancer cachexia. *Cancer Res.*, **51**, 2302-2306.

McGARRY JD AND FOSTER DW. (1976). An improved and simplified radioisotopic assay for the determination of free and esterified carnitine. *J. Lipid Res.*, **17**, 277-281.

MEMON RA, GRUNFELD C, MOSER AH AND FEINGOLD KR. (1993). Tumor necrosis factor mediates the effects of endotoxin on cholesterol and triglyceride metabolism in mice. *Endocrinology*, **132**, 2246-2253.

MOLEY JF, MORRISON SD AND NORTON JA. (1985). Insulin reversal of cancer cachexia in rats. *Cancer Res.*, **45**, 4925-4931.

NELSON KA, WALSH D AND SHEEHAN FA. (1994). The cancer anorexia-cachexia syndrome. *J. Clin. Oncol.*, **12**, 213-225.

NOGUCHI Y, VYDELINGUM JA, YOUNES RN, FRIED SK AND BRENNAN MF. (1991). Tumor-induced alterations in tissue lipoprotein lipase activity and mRNA levels. *Cancer Res.*, **51**, 863-869.

PARILLO JE. (1990). Septic shock in humans: Advances in the understanding of pathogenesis, cardiovascular dysfunction, and therapy. *Ann. Int. Med.*, **113**, 227-242.

PHILLIPS TM AND KIMMEL PL. (1994). High-performance capillary electrophoretic analysis of inflammatory citokines in human biopsies. *J. Chromatogr. B*, **656**, 259-266.

POPP MB, MORRISON SD AND BRENNAN MF. (1981). Total parenteral nutrition in a methylcholanthrene-induced rat sarcoma model. *Cancer Treat. Rep.*, **65**, 137-143.

SHERRY BA, GELIN J, FONG Y, MARANO M, WEI H, CERAMI A, LOWRY SF, LUNDHOLM KG AND MOLDAWER LL. (1989). Anticachectin/tumor necrosis factor-alpha antibodies attenuate development of cachexia in tumor models. *FASEB J.*, **3**, 1956-1962.

SMITH KL AND TISDALE MJ. (1993). Mechanism of muscle protein degradation in cancer cachexia. *Br. J. Cancer*, **68**, 314-318.

STALLION A, ZHANG FS, CHANCE WT, FOLEY-NELSON T AND FISCHER JE. (1991). Reversal of cancer cachexia in rats by cimaterol and supplemental nutrition. *Surgery*, **110**, 678-684.

STOVROFF MC, FRAKER DL AND NORTON JA. (1989). Cachectin activity in the serum of cachectic, tumor-bearing rats. *Arch. Surg.*, **124**, 94-99.

STRASSMANN G, FONG M, WINDSOR S AND NETA R. (1993). The role of interleukin-6 in lipopolysaccharide-induced weight loss, hypoglycemia and fibrinogen production, in vivo. *Cytokine*, **5**, 285-290.

TAKEYAMA J, TAKAGI D, MATSUO N, KITAZAWA Y AND TANAKA T. (1989). Altered hepatic fatty acid metabolism in endotoxicosis: effect of L-carnitine on survival. *Am. J. Physiol.*, **256**, E31-E38.

TRACEY KJ, FONG Y, HESSE DG, MANOGUE KR, LEE AT, KUO GC, LOWRY SF AND CERAMI A. (1987). Anti-cachectin/TNF monoclonal antibodies prevent septic shock during lethal bacteraemia. *Nature*, **330**, 662-664.

VON ROENN JH, ROTH EL AND CRAIG R. (1992). HIV-related cachexia, Potential mechanisms and treatment. *Oncology*, **49**, suppl 2, 50-54.

WELBOURN CRB AND YOUNG Y. (1992). Endotoxin, septic shock and acute lung injury: neutrophils, macrophages and inflammatory mediators. *Br. J. Surg.*, **79**, 998-1003.

WORTHLEY LIG, FISHLOCK RC AND SNOSWELL AM. (1983). Carnitine deficiency with hyperbilirubinemia, generalized skeletal muscle weakness and reactive hypoglycemia in a patient on long-term total parenteral nutrition: Treatment with intravenous L-carnitine. *J. Parenter. Enter. Nutr.*, **7**, 176-180.